# Synthesis and In Silico Evaluation of Piperazine-Substituted 2,3-Dichloro-5,8-dihydroxy-1,4-naphthoquinone Derivatives as Potential PARP-1 Inhibitors

**DOI:** 10.1021/acsomega.4c04915

**Published:** 2024-09-10

**Authors:** Ulviyye Nemetova, Pınar Si̇yah, Tuğçe Boran, Çiğdem Bi̇lgi̇, Mustafa Özyürek, Sibel Şahi̇nler Ayla

**Affiliations:** † Engineering Faculty, Department of Chemistry, Organic Chemistry, Istanbul University-Cerrahpaşa, 34320 Istanbul, Turkey; ‡ Department of Biochemistry, Faculty of Pharmacy, Bahcesehir University, 34353 Istanbul, Turkey; § Faculty of Pharmacy, Department of Pharmaceutical Toxicology, Istanbul University-Cerrahpaşa, 34500 Istanbul, Turkey; ∥ Faculty of Pharmacy, Department of Pharmacognosy, Istanbul University-Cerrahpaşa, 34500 Istanbul, Turkey; ⊥ Engineering Faculty, Department of Chemistry, Analytical Chemistry, Istanbul University-Cerrahpaşa, 34320 Istanbul, Turkey

## Abstract

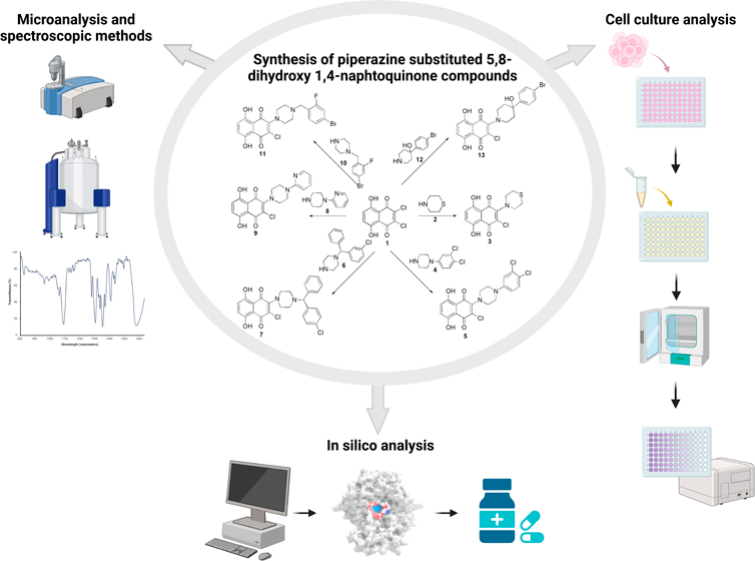

PARP-1 (poly(ADP-ribose)-polymerase 1) inhibitors are vital in synthetic lethality, primarily due to their specificity for PARP-1 over PARP-2 (PARP-1 > PARP-2). This specificity is crucial as it allows precise inhibition of PARP-1 in tumor cells with Breast Cancer 1 protein (BRCA1) or BRCA2 deficiencies. The development of highly specific PARP-1 inhibitors not only meets the therapeutic needs of tumor treatment but also has the potential to minimize the adverse effects associated with nonselective PARP-2 inhibition. In this study, a series of novel 2,3-dichloro-5,8-dihydroxy-1,4-naphthoquinone (DDNO) derivatives were synthesized, characterized, and evaluated regarding their PARP-1 inhibitory and cytotoxic activity. Compound 3 exhibited the highest cytotoxic potential against all cell lines, except for MDA-MB-231 cells. The inhibitory potential of these molecules against PARP-1 was evaluated through in silico molecular docking and molecular dynamics studies. Notably, compounds **5**, **9**, and **13** exhibited significant inhibitory activity in silico results, interacting with critical amino acids known to be important for PARP-1 inhibition during simulations. These compounds exhibited target-specific and strong binding profiles, with docking scores of −7.17, −7.41, and −7.37 kcal/mol, respectively, and MM/GBSA scores of −52.51, −43.77, and −62.87 kcal/mol, respectively. These novel compounds (DDNO derivatives) hold promise as potential PARP-1 inhibitors for the development of targeted therapeutics against cancer.

## 1 Introduction

PARP inhibitors (PARPi) have a remarkable effect in the treatment of tumors with homologous recombination repair (HR) deficiency. Specifically, PARPi is being used to target tumors with mutations in key HR genes (BRCA1 and BRCA2). Two research groups elucidated the concept of Synthetic Lethal (SL) interaction, linking the inhibition of PARP with mutations in BRCA1 or BRCA2, and proposed an innovative approach for treating patients with tumors carrying BRCA mutations.^1,2^ This genetic relationship between PARP and BRCA demonstrates synthetic lethality, where the loss of either gene alone is tolerable, but the simultaneous loss of both genes leads to cell death.^3^ The discovery that tumor cells with BRCA mutations were significantly more sensitive to PARPi—up to 1000 times more sensitive compared to BRCA wild-type cells (depending on the specific PARPi and experimental conditions)^2^ prompted the investigation of PARPi as monotherapies in clinical trials.^4,5^ PARP-1 inhibitors lead to synthetic lethality in tumor cells lacking either BRCA1 or BRCA2, or both, resulting in the eventual death of cancer cells.^6^ Goldberg et al.^7^ and Bryant et al.^5^ have shown that the genetic depletion of PARP-1 is adequate to trigger the demise of BRCA1-deficient and BRCA2-deficient tumor cells, respectively. Hence, the development of highly specific PARP-1 inhibitors can fulfill the needs of tumor therapy while potentially minimizing the adverse effects associated with PARP-2 inhibition. Due to the considerable similarity in the catalytic domains of PARP-1 and PARP-2, achieving a high level of selectivity inhibition poses challenges; however, the synthesis of new molecules with high selectivity and PARP-1 inhibition activity has gained a crucial role in medicinal chemistry.^8^

The synthesis of piperazine (hexahydropyrazine diethylenediamine) and its derivatives, which have a six-membered ring structure containing two nitrogen atoms at the C-1 and C-4 positions, has increased significantly in recent years. One of the main reasons for this increase is the important pharmacological effects of these structures.^1,9−11^ Several piperazine derivatives are being used for clinical application. Indinavir (Crixivan), which functions as an HIV protease inhibitor, can be given as a most known example. A series of piperazine derivatives were just synthesized and tested for potential antidepressant activity to 5-hydroxytryptamine (serotonin, 5-HT1A) receptor (5-HT1AR).^12^ Another important pharmacophore, quinone, has a big spectrum of biological activities such as antibacterial, antiviral, anticancer, and antifungal.^13−16^ Quinone derivatives have a wide range of uses in areas such as cosmetics, drugs, and paint industry.^17^ The structures of this kind of compounds can also be found in natural products such as juglone, lawsone, menadion, lapachol, or important compounds that can be obtained synthetically.^18^ They play important roles in metabolic pathways via electron transport chains. Among the quinones, 2,3-dichloro-5,8-dihydroxy-1,4-naphthoquinone (DDN) has an important place, and even just one molecule has dual potential as an inhibitor of acetylcholinesterase (AChE) and Aβ42 aggregation.^19^ Although there are a limited number of studies in the literature on the hydroxylated 1,4-naphthoquinone compound, it is seen that the hydroxyl groups on the C5 and C8 carbons cause an increase in biological properties.^20^

As a continuation of our work on quinones, a series of novel piperazine derivatives with a hydroxylated quinone moiety were synthesized. The structures of novel molecules were elucidated using spectroscopic methods such as FT-IR, ^1^H NMR, ^13^C NMR, and UV–vis. The cytotoxic potentials of compounds **3**, **5**, **7**, **9**, **11**, and **13** were investigated with MTT [3-(4,5-dimethylthiazol-2-yl)-2,5-diphenyltetrazolium bromide] test. Furthermore, all synthesized molecules were subjected to in silico analyses including molecular docking studies, short-, medium-, and long-term molecular dynamics simulations at various time intervals and MM/GBSA analysis to explore their potential as PARP-1 inhibitors. These molecules were targeted against both PARP-1 and PARP-2. Lastly, molecules that exhibited high affinity and stability specifically to PARP-1 but exhibited weak binding to PARP-2 were identified as potential inhibitor drug candidates specific to the PARP-1 target.

## 2 Experimental Section

### 2.1 General Procedure

A solution of 2,3-dichloro-5,8-dihydroxy-1,4-naphthoquinone (**1**) and the corresponding piperazine compound in dichloromethane (DCM) was added to a solution of Na_2_CO_3_ in DCM and stirred for 30–45 min at room temperature. The reaction mixture immediately changed color and was kept at room temperature overnight and then filtered. The organic layer was dried with Na_2_SO_4_ and filtered. The final compounds were purified by a chromatographic column. The amounts of reactants and eluent solution ratios were mentioned in the experimental details.

#### 2.1.1 2-Chloro-5,8-dihydroxy-3-thiomorpholinonaphthalene-1,4-dione (**3**)

**3** was synthesized from 2,3-dichloro-5,8-dihydroxy-1,4-naphthoquinone (**1**) (0.1 g, 0.38 mmol) and thiomorpholine (**2**) (0.04 g, 0.38 mmol) by use of the general procedure.

(**3**): Dark violet solid. m.p.: 245–246 °C. Yield: 67% (0.08 g). *R*_f_: 0.23 [CHCl_3_]. IR(ATR) ν (cm^–1^) = 2997, 2898, 2861 (−CH), 1683 (C=O), and 1605 (C=C). ^1^H NMR (499.74 MHz, CDCl_3_): δ = 12.53, 12.51 (s, 2H, −OH), 3.66 (t, *J* = 4.88 Hz, 2H, –NCH_2_), 2.72 (t, *J* = 4.88 Hz, 2H, –SCH_2_), 7.06–7.08, 7.12–7.14 (m, 2H, CH_arom_). ^13^C NMR (125.66 MHz, CDCl_3_): δ = 52.45 (–NCH_2_), 159.69, 156.99, 155.50, 149.93, 141.40, 129.64, 128.62, 126.90 (C_arom_, CH_arom_), 26.70 (-SCH_2_), 52.45 (–NCH_2_), 183.20,180.05 (C=O). UV–vis (C_2_H_5_OH) λ(logε) = 538(2.93), 272(3.10), 210(3.37), and 196(2.90) nm. UV–vis (CHCl_3_) λ(logε) = 865(0.55), 709(0.3), 523(2.92), 275(3.15) nm. MS(+ESI): 326.1 [M + H]^+^. C_14_H_12_ClNO_4_S (M = 325.77g/mol), Calcd C, 51.62; H, 3.71; N, 4.30; S, 9.84. Found C, 51.55; H, 3.42; N, 4.22; S, 8.99.

#### 2.1.2 2-Chloro-3-(4-(3,4-dichlorophenyl)piperazin-1-yl)-5,8-dihydroxynaphthalene-1,4-dione (**5**)

**5** was synthesized from 2,3-dichloro-5,8-dihydroxy-1,4-naphthoquinone (**1**) (0,05 g, 0.19 mmol) and 1-(3,4-dichlorophenyl)piperazine (**4**) (0.044 g, 0.19 mmol) by the use of the general procedure.

(**5**): Dark purple solid. m.p.: 226–227 °C. Yield: 75% (0.065 g). *R*_f_: 0.23 [CH_2_Cl_2_]. IR(ATR) ν(cm^–1^) = 2955, 2916, 2887, 285 (−CH), 1639 (C=O), 1595 (C=C). ^1^H NMR (499.74 MHz, CDCl_3_): δ = 12.64, 12.26 (s, 2H, −OH), 3.28 (t, *J* = 4.88 Hz, 2H, N–CH_2_), 3.71 (t, *J* = 4.88 Hz, 2H, N–CH_2_), 1.48 (*J* = 4.88, 4H, N–CH_2_) 6.95–7.26 (m, 5H, C_arom_, CH_arom_). ^13^C NMR (125.66 MHz, CDCl_3_): δ = 50.01, 48.80 (–NCH_2_), 166.34, 157.48, 155.89, 149.13, 131.90, 129.52, 129.19, 127.68, 127.23, 122.64, 116.81, 114.81, 110.30, 109.37, 109.28, 108.88, 76.15, 75.89, 75.64, 75.46 (C_arom_, CH_arom_), 183.5, 180.31 (C=O). UV–vis (C_2_H_5_OH) λ (logε) = 518(2.18), 265(2.56), 210(2.76), 200(2.65) nm. UV–vis (CHCl_3_) λ (logε) = 892(0.62), 872(0.76), 846(0.97), 812(0.91) nm. MS(+ESI): 453.2 [M + H]^+^. C_20_H_15_Cl_3_N_2_O_4_ (M = 453.7 g/mol). Calcd C, 52.95; H, 3.33; N, 6.16. Found C, 52.87; H, 3.18, N, 6.23.

#### 2.1.3 2-Chloro-3-(4-((4-chlorophenyl)(phenyl)methyl)piperazin-1-yl)-5,8-dihydroxynaphthalene-1,4-dione (**7**)

**7** was synthesized from 2,3-dichloro-5,8-dihydroxy-1,4-naphthoquinone (**1**) (0.1 g, 0.38 mmol) and 1-(4-Chlorobenzhydryl)piperazine (**6**) (0.1 g, 0.38 mmol) by using the general procedure.

****(**7**): Violet solid. m.p.: 230–231 °C. Yield: 52% (0.10 g). *R*_f_: 0.75 [CH_2_Cl_2_]. IR(ATR) ν(cm^–1^) = 2960, 2924, 2882, 2819 (−CH), 1637 (C=O), 1558 (C=C). ^1^H NMR (499.74 MHz, CDCl_3_): δ = 13.90, 12.96 (s, 2H, −OH), 2.49 (s, 2H, -NCH_2_), 3.52 (m, 4H, –NCH_2_), 3.54 (t, *J* = 8.79 Hz, 2H, –NCH_2_), 7.13–7.68 (m, 11H, C_arom_, CH_arom_). ^13^C NMR (125.66 MHz, CDCl_3_): δ = 51.54, 50.83 (-NCH_2_), 157.23, 155.55, 149.3, 140.56, 139.75, 131.75, 130.35, 128.97, 128.81, 128.37, 128.04, 127.74, 27.68, 127.52, 127.3, 126.85, 126.69, 126.46, 126.33, 121.33, 121.28 ve 110.38 (C_arom_, CH_arom_), 183.71, 180.35 (C=O). UV–vis (C_2_H_5_OH) λ (logε) = 521 (3.09), 378 (2.18), 273 (3.36), 229 (3.41) nm. UV–vis(CHCl_3_) λ (logε) = 873 (2.17), 827 (2.29), 790 (2.29), 719(2.29) nm. MS(+ESI): 509.0 [M + H]^+^ C_27_H_22_Cl_2_N_2_O_4_ (M = 509.38 g/mol). Calcd C, 63.66; H, 4.35; N, 5.50; Found C, 63.41; H, 4.18; N, 5.27.

#### 2.1.4 2-Chloro-5,8-dihydroxy-3-(4-(pyridin-2-yl)piperazin-1-yl)naphthalene-1,4-dione (**9**)

**9** was synthesized from 2,3-dichloro-5,8-dihydroxy-1,4-naphthoquinone (**1**) (0,1 g, 0.38 mmol) and 1-(2-pyridyl)piperazine (**8**) (0.06 g, 0.38 mmol) by the general procedure.

(**9**): Purple oil. Yield: 68% (0.11 g). *R*_f_: 0.33 [CH_2_Cl_2_]. IR (ATR) ν(cm^–1^) = 2959, 2916, 2849 (−CH), 1644 (C=O), and 1592 (C=C). ^1^H NMR (499.74 MHz, CDCl_3_): δ = 12.66, 12.25 (s, 2H, −OH), 3.67 (s, 4H, –NCH_2_), 6.61–8.14 (m, 6H, C_arom_,CH_arom_). ^13^C NMR (125.66 MHz, CDCl_3_): δ = 50.05, 44.96 (-NCH_2_), 157.88, 157.16, 155.52, 149.16, 146.72, 136.40, 128.87, 126.84, 121.77, 112.65 (C_arom_, CH_arom_), 183.45, 180.14 (C=O). UV–vis (C_2_H_5_OH) λ (logε) = 519(3.84), 270(4.14), 253(4.21), 208(4.29) nm. UV–vis (CHCl_3_) λ (logε) = 793(2.38), 524(4.05), 259(4.42), 239(4.35) nm. MS(+ESI): 386.3 [M + H]^+^. C_19_H_16_ClN_3_O_4_ (M = 385.80 g/mol). Calcd C, 59.15; H, 4.18; N, 10.89. Found C, 59.22; H, 4.38; N, 10.55.

#### 2.1.5 2-(4-(4-Bromo-2-fluorobenzyl)piperazin-1-yl)-3-chloro-5,8-dihydroxynaphthalene-1,4-dione (**11**)

**11** was synthesized from 2,3-dichloro-5,8-dihydroxy-1,4-naphthoquinone (**1**) (0,1 g, 0.38 mmol) and 1-(4-bromo-2-fluorobenzyl)piperazine (**10**) (0,1 g, 0.38 mmol) by the general procedure.

(**11**): Violet oil. Yield: 48% (0.18 g). *R*_f_: 0.50 [CHCl_3_]. IR(ATR) ν (cm^–1^) = 3664 (−OH), 2987, 2968, 2924, (−CH), 1635 (C=O), 1542 (C=C). ^1^H NMR (499.74 MHz, CDCl_3_): δ = 12.65, 12.23 (s, 2H, OH), 2.03 (s, 2H-NCH_2_), 3.55 (d, 4H-NCH_2_), 4.55 (s, 2H-NCH_2_), 4.83 (s, 2H-NCH_2_), 6.97–8.02 (m, 5H, CH_arom_). ^13^C NMR (125.66 MHz, CDCl_3_): δ = 53.95, 52,57, 51.63 (–NCH_2_), 166.81, 161.70, 159.69, 143.21,130.92, 130.76, 128.99, 128.67, 118.80, 118.60, 115.47, 110.89 (C_arom_, CH_arom_), 180.82 (C=O).UV–vis (C_2_H_5_OH) λ (logε) = 786(2.34), 520(3.61), 275 (3.89), 269(3.89) nm. UV–vis (CHCl_3_) λ (logε) = 846(1.69), 826(1.75), 798(1.83), 718(1.88) nm. MS(+ESI): 497.1 [M + H]^+^. C_21_H_17_BrClFN_2_O_4_ (M = 495.72g/mol). Calcd C, 50.88; H, 3.46; N, 5.65. Found C, 50.58; H, 3.51; N, 5.90.

#### 2.1.6 2-(4-(4-Bromophenyl)-4-hydroxypiperidin-1-yl)-3-chloro-5,8-dihydroxynaphthalene-1,4-dione (**13**)

**13** was synthesized from 2,3-dichloro-5,8-dihydroxy-1,4-naphthoquinone (**1**) (0,1 g, 0.38 mmol) and 4-(4-bromophenyl)-4-piperidinol (**12**) (0,1 g, 0.38 mmol) by use of the general procedure.

****(**13**): Violet solid. m.p.: 241–242 °C. Yield: 49% (0.09 g). *R*_f_: 0.50 [CH_2_Cl_2_]. IR(FTR); ν (cm^–1^) = 3493 (−OH), 2954, 2918, 2868 (−CH), 1693 (C=O), 1603 (C=C). ^1^H NMR (499.74 MHz, CDCl_3_): δ = 12.73, 12.31 (s,–OH), 4.15 (m, 2H, –NCH_2_), 3.75 (m, 2H, –NCH_2_), 2.25 (m, 2H, −CH_2_), 3.04 (m, 2H, −CH_2_), 7.15–7.66 (m, 6H, C_arom_, CH_arom_). ^13^C NMR (125.66 MHz, CDCl_3_): δ = 46.96, 44.70 (–NCH_2_), 166.65, 164.86, 157.29, 155,63,149.97, 145.53, 131.34, 130.54, 129.72, 128.37, 127.68, 1255.29 (C_arom_, CH_arom_), 46.96, 44.70 (–NCH_2_), 29.25, 30.81, 38.11 (−CH_2_), 180.40, 183.81 (C=O). UV–vis (C_2_H_5_OH) λ (logε) = 523(3.69), 275(4.01), 206(4.30), 297(4.01) nm. UV–vis (CHCl_3_) λ (logε) = 893(1.73), 846(3.70), 798(1.74), 760(1.85) nm. MS(+ESI): 480.01[M + H]^+^. C_21_H_17_BrClNO_5_ (*M* = 478.72g/mol). Calcd C, 52.69; H, 3.58; N, 2.93. Found C, 52.59; H, 3.45; N, 2.88.

### 2.2 In Silico Studies

#### 2.2.1 Preparation of the Piperazine-Substituted 2,3-Dichloro-5,8-dihydroxy-1,4-naphthoquinone Derivative Compounds for Molecular Docking

The ligands and proteins used for docking were prepared with high precision and accuracy.^21^ Initially, the compounds were sketched using the 2D Sketcher of Maestro. To utilize the ligands in docking studies, the 2D structures of the compounds were transformed into optimized and energetically minimized 3D structures using Maestro’s LigPrep module.^22,23^ The optimization process employed the OPLS3e force field.^24^

#### 2.2.2 Preparation of PARP-1 Protein for Molecular Docking

Preparation of the proteins for molecular docking followed the procedures outlined in our previous study. Briefly, X-ray crystal structure for 7ONT(25) was obtained from the Protein Data Bank. The structure was simplified by eliminating the B chain. Only the “A” chain of the cocrystallized ligand 7ONT protein complex was kept from the human PARP-1 structure, while other ligands were discarded. Mutations were reverted to the wild-type sequence. The protein preparation studies were carried out using protein preparation module of Schrodinger’s Maestro Molecular modeling package. The OPLS3e force field was employed for restrained minimization, with a heavy atom convergence of 0.3 Å. Disulfide bonds were formed, and hydrogens and bond orders that were initially absent were incorporated using the Prime module.^26^ Protonation states were determined at a physiological pH of 7.4 using PROPKA.^27^ Protonated residues underwent hydrogen bond optimization. Next, the systems were assigned by PROPKA with consideration of a physiological pH, followed by thorough energy minimization using the OPLS3e force field. The crystallized ligand binding sites on the target proteins were then determined as grid boxes with the removal of any ions or small elements introduced for crystallization purposes. Upon successful completion of these preparations, the docking studies were conducted as per our previous publications.^28,29^

#### 2.2.3 Grid Box Generation and Molecular Docking Studies

The determination of the crystallized ligand binding site on the target protein involved identifying the center of the grid box. This process was carried out as part of grid generation in the receptor grid generation panel aimed at representing the active binding pocket of the protein for subsequent docking. The receptor’s grid generation was initiated by selecting the ligand from the prepared protein, ensuring the exclusion of the ligand from both grid generation calculations and ligand–receptor docking. The final step involved implementing the receptor grid generation with default settings, incorporating constraints on rotatable groups, and excluding volume. This was achieved by scaling the van der Waals radius with a scaling factor of 1.0 and employing a cutoff for the partial charge. The piperazine-substituted 2,3-dichloro-5,8-dihydroxy-1,4-naphthoquinone derivative compounds were subsequently subjected to molecular docking with PARP-1 and PARP-2 proteins. To accomplish this, we employed Maestro’s Glide module with standard accuracy settings.^30^

#### 2.2.4 Molecular Dynamics Simulations

To investigate the dynamic behavior and stability of the complexes, molecular dynamics (MD) simulations were carried out in accordance with the methodology outlined in our previous publication.^31^ All simulations were conducted utilizing Desmond software Bowers. In order to mimic the physiological environment, the grid boxes were filled with TIP3P water molecules. The neutralization of the systems was achieved by introducing 0.15 M sodium and chloride ions. Throughout the simulations, a constant temperature of 310 K and a pressure of 1.01325 bar were maintained by using the Hoover thermostat and Martyna-Tobias-Klein protocols. Complexes underwent a meticulous simulation study spanning 10 and 100 ns, enabling a comprehensive analysis of their behavior and interactions. The complexes with the highest scores were subsequently subjected to further examination regarding their free binding energy through MM/GBSA analysis using the Schrodinger Prime module.^26^

#### 2.2.5 ADME Analysis and Therapeutic-QSAR Models

To determine the ADME (absorption, distribution, metabolism, and excretion) properties and therapeutic activities of the molecules, we employed the MetaCore/MetaDrug platform from Clarivate Analytics was employed. Through this platform, comprehensive analyses were conducted on all of the molecules included in the study. Specifically, these molecules were evaluated in terms of therapeutic activity using 25 diverse common disease QSAR (quantitative structure–activity relationship) models. To ensure the reliability and robustness of the predictive models, extensive validation was carried out. This involved assessing the models’ performance using key parameters such as sensitivity, specificity, accuracy, and the Matthews correlation coefficient (MCC), allowing for an accurate assessment of their quality. A threshold value of 0.5 was set.

### 2.3 Cytotoxicity Assay (In Vitro Studies)

MCF7 (ATCC, HTB22), A549 (ATCC, CCL-185), and SH-SY5Y (ATCC, CRL-2266) (DKFZ, CLS 300493) cells were cultured in Dulbecco’s modified Eagle’s medium (DMEM) supplemented with 10% fetal bovine serum (FBS) and 1% antibiotic/antimycotic solution. HepG2 (ATCC, HB-8065) and MDA-MB-231 (ATCC, HTB-26) cells were maintained in Roswell Park Memorial Institute (RPMI) medium containing 10% FBS and 1% antibiotic/antimycotic solution. The cell culture medium was changed every 2–3 days. The cells were subcultured when they reached 60–70% confluence.

The cytotoxic potentials of compounds **3**, **5**, **7**, **9**, **11**, and **13** were investigated with the MTT [3-(4,5-dimethylthiazol-2-yl)-2,5-diphenyltetrazolium bromide] test. The cells were seeded into a 96-well plate at a density of 1 × 10^4^ cells per well and incubated overnight at 37 °C and 5% CO_2_ to be attached. The cells were exposed to the chemicals at different concentrations and incubated for 24 h at 37 °C and 5% CO_2_. Then, 20 μL of MTT solution (0.5 mg/mL) was added to each well. After 3 h incubation, the cell culture supernatant was discarded and 100 μL of dimethyl sulfoxide was added to each well to dissolve formazan crystals. Optical density (OD) was measured with a microplate reader (Biotek, Germany) at 590 nm. The cell viability was determined as a percentage of the control group, which was exposed to 1% DMSO-containing medium, and the half-maximal inhibitory concentration (IC_50_) value was calculated. The cells exposed to 1% Triton X-100 were used as a positive control. The cytotoxic potentials of the chemicals were expressed using IC_50_ values.

## 3 Results and Discussion

### 3.1 Synthesis of Compounds

**Figure 1:**
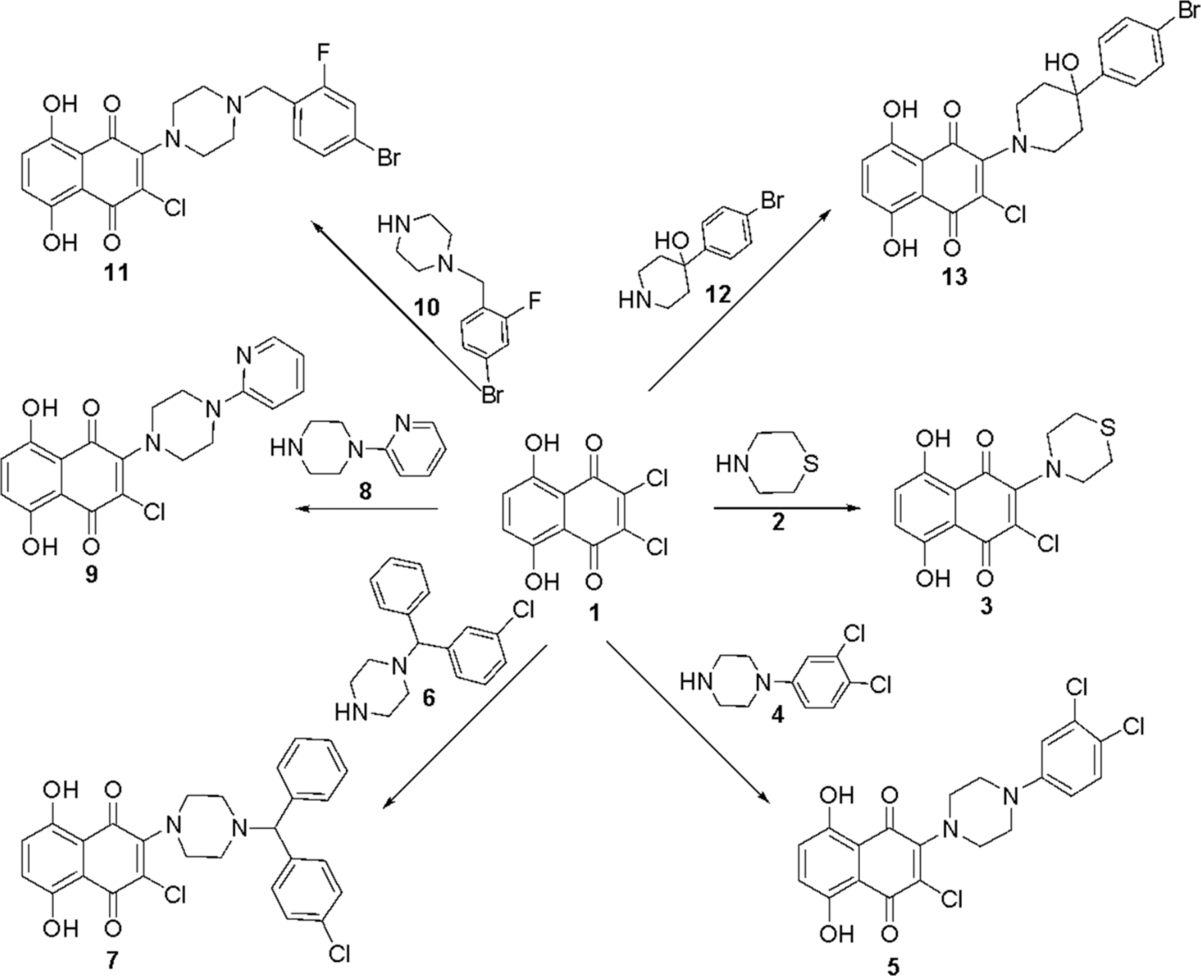
Synthetic pathway of piperazine-substituted 5,8-dihydroxy 1,4-naphthoquinone compounds.

The synthesis of a series of novel cyclic amine-substituted 2,3-dichloro-5,8-dihydroxy-1,4-naphthoquinone focused on the replacement of one of the chlorine atoms on the C2 carbon in the quinone pharmacophore. The structures of the novel compounds are outlined in Figure 1.

**Figure 2:**
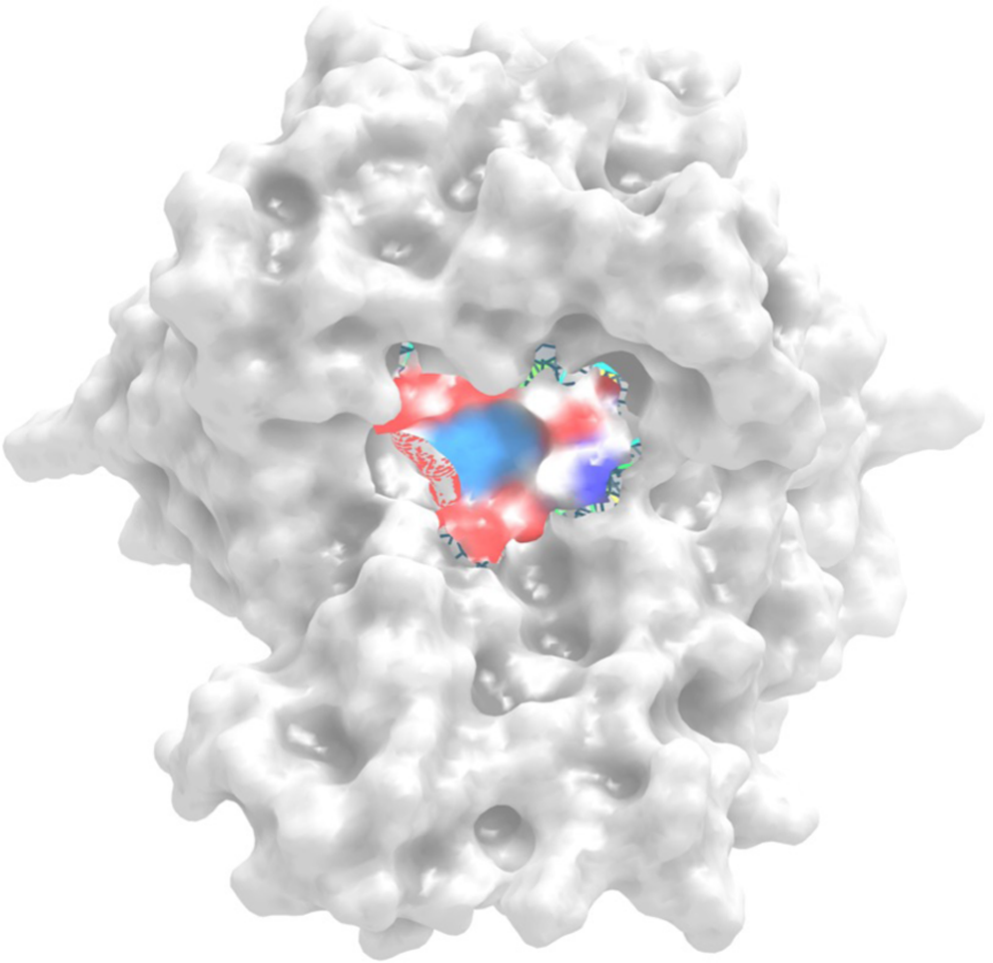
Three-dimensional structure of the prepared PARP-1 protein (PDB ID: 7ONT) with the active region highlighted.

The mass spectra of thiomorpholine-substituted compound **3** in positive-ion mode for ESI confirmed the proposed structures; the protonated molecular ion was observed at *m*/*z* 326 (100%). The carbon signals of the (–NCH_2_) carbons of compound **3** were at 52.45 ppm in the ^13^C NMR. The carbonyl group of compound **5** gave a characteristic peak at 1639 cm^–1^ of compound **5** in the IR spectra and hydroxyl signals at 12.64 and 12.26 ppm as singlets at the ^1^H NMR. The protonated molecular ion peaks of piperazine-substituted derivatives compounds **7** and **9** were detected at *m*/*z* 509 and 386 (100%), respectively. 2,3-Dichloro-5,8-dihydroxy-1,4-naphthoquinone, compound **1**, was treated with compound **10**, and novel *N*-substituted compound **11** was achieved. The mass spectra of piperazinyl-substituted compound **11** in positive-ion mode for ESI confirmed the proposed structures; the protonated molecular ion was observed at *m*/*z* 497 (100%). Under the same reaction conditions, compound **13** was obtained from the reaction of compounds **1** and **12**. The hydroxyl groups of compound **13** gave a characteristic peak at 3570 cm^–1^ in the IR spectra. Synthesized piperazine-substituted 2,3-dichloro-5,8-dihydroxy-1,4-naphthoquinone derivative compounds were targeted to the active region in the three-dimensional structure of the prepared PARP-1 protein (Figure 2).

### 3.2 In Silico Analysis

Based on the docking score table, it was observed that compounds **3**, **5**, **9**, and **13** exhibited the best targeting with PARP-1, with docking scores ranging from −6.52 to −7.41 kcal/mol and ligand efficiencies between −0.25 and −0.31. Among the six molecules studied, the top four molecules showed significant clustering in both docking score and ligand efficiency, while the subsequent two molecules demonstrated a noticeable decrease. The docking scores of the first 5 molecules for PARP-1 protein were consistently lower (at least 1 kcal/mol lower) in comparison to their docking against the PARP-2 protein. This suggests that compounds **3**, **5**, **9**, and **13** could potentially serve as specific small-molecule inhibitors for PARP-1. Noteworthy is the observation that the docking scores of known inhibitor drugs on the market employed as reference compounds in this study closely aligned with those of the top 5 molecules, falling within the range of −5.23 to −7.43 kcal/mol. However, these reference molecules also possessed a targeting potential for PARP-2 (Table 1). None of them exhibited a difference of even 1 kcal/mol between PARP-1 and PARP-2, with niraparib and olaparib even binding more strongly to PARP-2. This indicated that the known drugs are not selective for PARP-1, while newly synthesized piperazine-substituted 2,3-dichloro-5,8-dihydroxy-1,4-naphthoquinone derivative compounds have the potential to be better candidates with specific molecular targeting for PARP-1 protein.

**Table 1:** Docking and Ligand Efficiency Scores for the Ligands at the Binding Sites of PARP Proteins

	PARP-1 (7ONT)		PARP-2 (4TVJ)	
ligand name	docking score (kcal/mol)	ligand efficiency	docking score (kcal/mol)	ligand efficiency
compound **9**	–7.41	–0.274	–5.82	–0.215
compound **13**	–7.37	–0.254	–6.05	–0.209
compound **5**	–7.17	–0.247	–4.00	–0.138
compound **3**	–6.52	–0.310	–5.46	–0.260
compound **7**	–2.27	–0.065	–5.72	–0.163
compound **11**	–2.18	–0.073	–6.59	–0.220
nicotinamide	–7.43	–0.826	–7.33	–0.814
rucaparib	–7.36	–0.307	–6.51	–0.271
niraparib	–7.11	–0.296	–7.58	–0.316
olaparib	–5.23	–0.163	–13.94	–0.436

**Figure 3:**
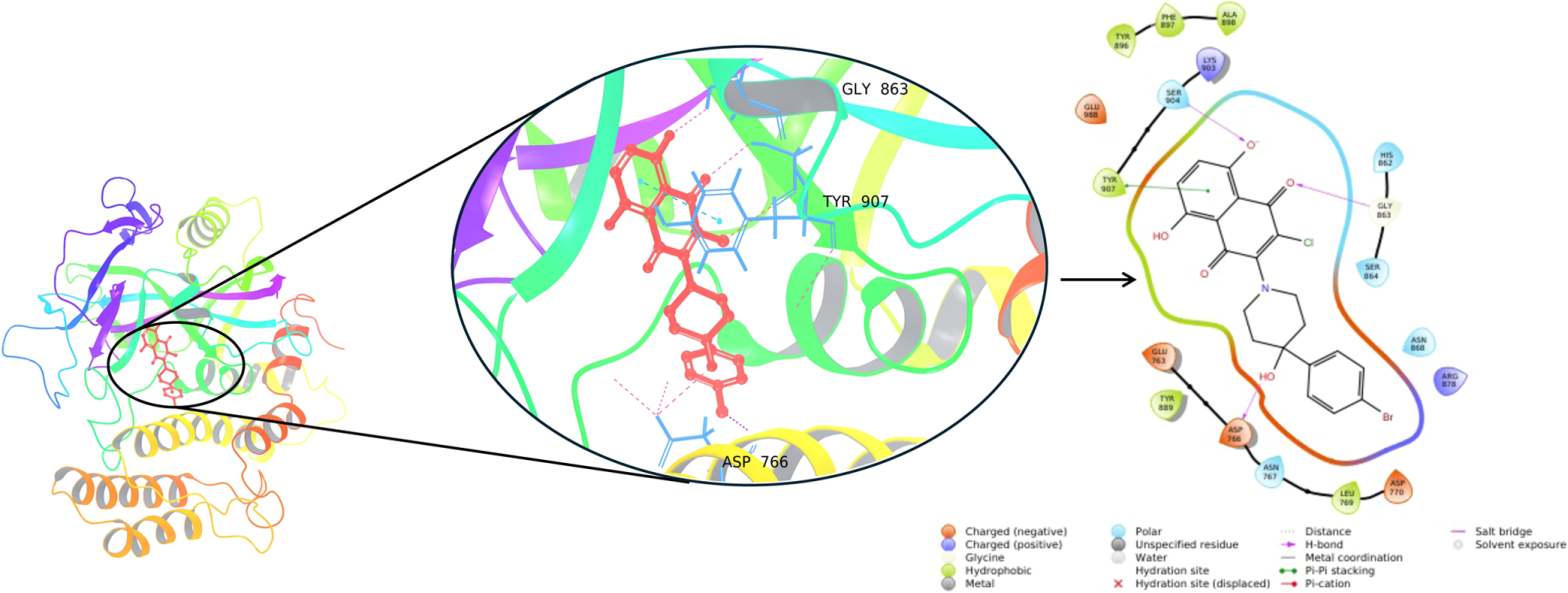
Three-dimensional ligand interaction diagram of compound **13** at the PARP-1 binding site.

The top docking conformation of compound **13** within the three-dimensional structure of the PARP-1 protein for the top-scoring is presented, along with the interactions (Figure 3). Key amino acids involved in these interactions include Gly863, Tyr907, and Asp766. The importance of these residues in the literature, where they are identified as critical amino acids in PARP docking interaction studies, is consistent with our findings.^32−35^

A map of the interactions that compound **13** engaged in the binding region of PARP-1 during a 100 ns simulation was generated. The most prominent interactions occurred with Gly863, Asp766, and Tyr907 amino acids, which aligned with our docking results and further validated the importance of these interactions in various studies.

In this study, MD simulations of 10 and 100 ns duration were conducted for all molecules, followed by MM/GBSA analyses. These analyses revealed that Olaparib exhibited the highest binding affinity with a score of −66.70 kcal/mol, indicating strong interaction with PARP-1. However, Olaparib’s higher affinity toward PARP-2 suggested its lack of specificity toward PARP-1. Additionally, compound **13** displayed significant binding affinity, with a score of −62.87 kcal/mol. Therefore, according to the MM/GBSA calculations after the 100 ns MD simulations, compound **13** emerged as the best molecule with high affinity toward PARP-1, showing specificity toward this target. The substantial energy difference in binding affinity between PARP-1 and PARP-2 upon compound **13**’s binding further confirms its specificity toward PARP-1.

Following compound **13**, Niraparib was identified with a score of −55.42 kcal/mol in the ranking of MM/GBSA scores against PARP-1. However, Niraparib lacks specificity for PARP-1 and has potential binding affinity toward PARP-2. Furthermore, among the synthesized molecules, compound **5** emerged as the second-best drug candidate. It showed strong affinity toward PARP-1 while displaying low affinity toward PARP-2, making it a target-specific drug candidate. On the contrary, Rucaparib exhibited better binding toward PARP-2 than PARP-1, leading to an undesired outcome. Compound **9** showed slightly better affinity toward PARP-1 than PARP-2, resulting in a positive outcome. Compound **7** and compound **11** were eliminated from the study as they showed better binding toward PARP-2 rather than PARP-1.

The MM/GBSA score for Talazoparib was not available (N/A), indicating that its binding affinity could not be determined in this context. In conclusion, compounds **13**, **5**, and **9** were identified as superior ligands with the strongest binding affinities toward PARP-1 among the compounds studied. These molecules were identified as PARP-1-specific inhibitors with a promising potential for use in cancer treatment (Table 2).

**Table 2:** MM/GBSA Scores of Ligand-Protein Complexes after 100 ns MD Simulations

PARP-1		PARP-2	
ligand name	MM/GBSA score (kcal/mol)	ligand name	MM/GBSA score (kcal/mol)
*olaparib*^a^	–66.70	*olaparib*	–99.96
compound **13**^b^	**–62.87**	*rucaparib*	–63.13
*niraparib*	–55.42	*niraparib*	–62.31
compound **5**	**–52.51**	*talazoparib*	–61.40
*rucaparib*	–49.67	compound **7**	–55.62
compound **9**	**–43.77**	compound **11**	–46.68
compound **7**	–36.45	compound **13**	–46.40
compound **11**	–33.90	compound **9**	**–40.37**
*nicotinamide*	–30.35	compound **5**	–38.55
*talazoparib*	N/A	*nicotinamide*	–38.54

a Italicized entries represent reference PARP inhibitor drugs.

b The synthesized compounds that showed promising results as PARP-1 over PARP-2 selective inhibitors are indicated in bold.

**Figure 4:**
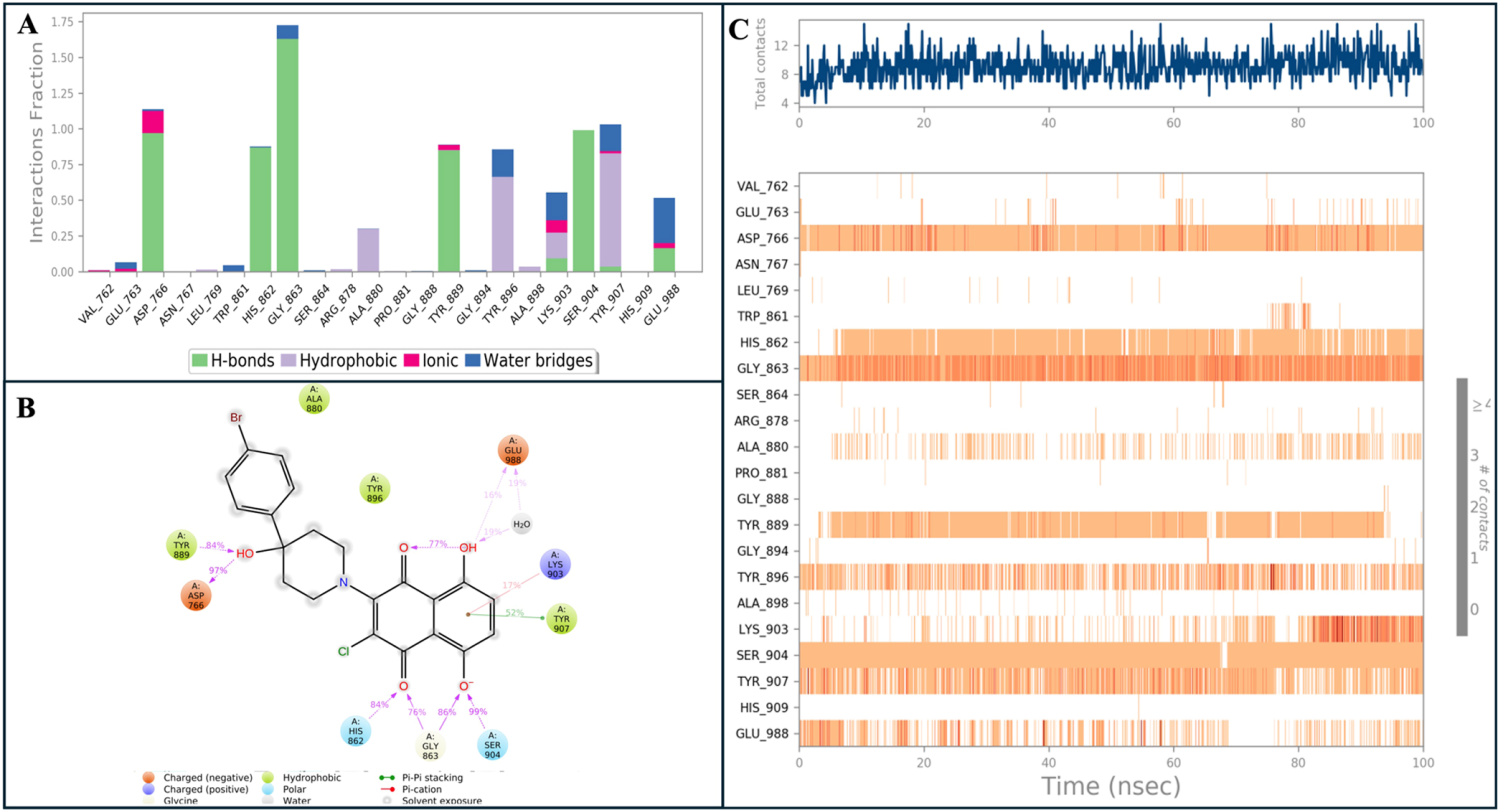
(A) Analysis of the interactions between binding pocket residues of compound **13** throughout the MD simulations. (B) Two-dimensional ligand interaction diagram of compound **13** at the PARP-1 binding site. (C) Interaction percentages of residues in the binding pocket of PARP-1 with compound **13** during the MD simulations. The findings present statistical outcomes based on 100 trajectory frames collected over 10 ns MD simulations.

In the analysis of simulation interactions, compound **13** exhibited pronounced interactions with Gly863, Asp766, and Tyr907 amino acids, forming stable hydrogen bonds that persisted throughout the entire simulation. Each interaction site also established water bridges, and an ionic bond with Asp766 was observed. Additionally, compound **13** engaged in hydrogen bonding with His862, Tyr889, Ser904, and Glu988. Hydrophobic interactions were realized with Ala880, Tyr896, Lys903, and Tyr907. Ionic bonds and water bridges with Lys903 and Gly988 were also discerned. Over 99% of the simulation duration, it interacted with Ser904; 97% with Asp766; 86% with Gly863; 84% with His862 and Tyr889; and 52% with Tyr907 (Figure 4).

**Figure 5:**
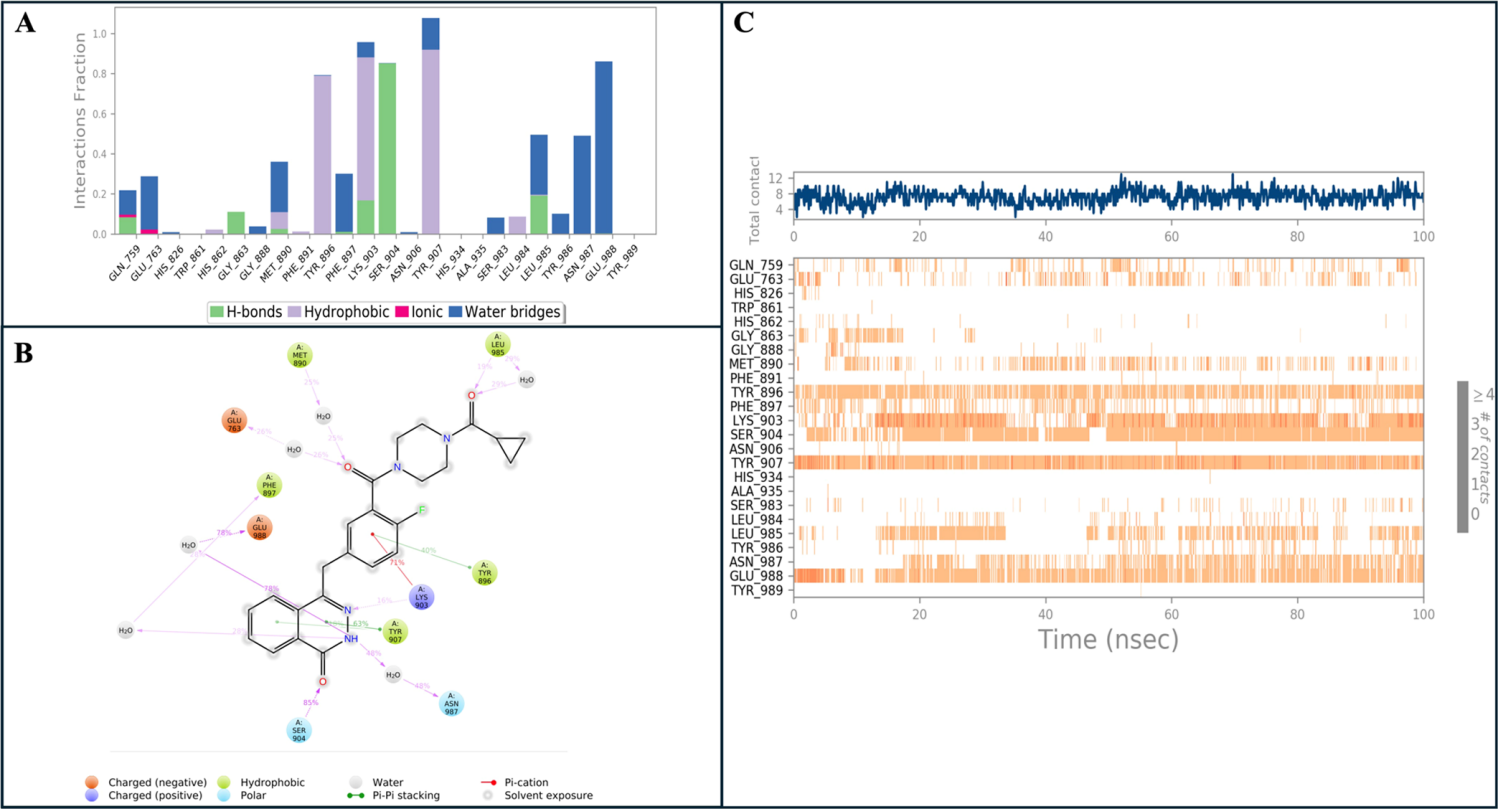
(A) Analysis of the interactions between binding pocket residues of Olaparib and PARP-1 throughout the MD simulations. (B) Two-dimensional ligand interaction diagram of Olaparib at the PARP-1 binding site. (C) Interaction percentages of residues in the binding pocket of PARP-1 with Olaparib during the MD simulations. The findings present statistical outcomes based on 100 trajectory frames collected over 10 ns MD simulations.

As the reference compound, Olaparib demonstrated the highest interactions with Lys903, Ser904, and Tyr907 amino acids, forming stable hydrogen bonds that remained consistent throughout the simulation. Noteworthy interactions with Tyr896 and Glu988 were also observed. Additionally, interactions with Met890 and Leu985 were noted. Olaparib interacted with Ser904 for 85% of the simulation time, with Lys903 for 71%, and with Tyr907 for 63% (Figure 5).

According to a recent study,^33^ the comparison of PARPi structures bound to PARP-1/2 revealed three critical interactions involving Gly863, Ser904, and Tyr907. These interactions are crucial for PARP-1’s interaction with inhibitors and underscore its significance in PARPi binding.

(1) Gly863 (Gly429 in PARP-2) forms two hydrogen bonds with the bi- or tricyclic ring system of each inhibitor in PARP-1, where Gly863’s amide nitrogen acts as an H-bond donor and the carbonyl oxygen serves as an H-bond acceptor.
(2) Ser904 (Ser470 in PARP-2) acts as an H-bond donor to a carbonyl in/on the bi- or tricyclic ring system of each inhibitor, contributing to the bidentate interaction of Gly863 and forming the basis for the inhibitor’s mimicry of nicotinamide.
(3) Tyr907 (Tyr473 in PARP-2) engages in a π–π interaction with the aromatic bi- or tricyclic ring of each inhibitor, despite not forming a similar interaction with the nicotinamide ring. This interaction significantly enhances PARPi’s affinity compared to nicotinamide and plays a vital role in binding inhibitors with larger aromatic ring structures, such as olaparib, more effectively than those with smaller aromatic rings, like veliparib.

In another study,^32^ it has been demonstrated that the binding of 7-MG to the active site of PARP-1 is mediated by hydrogen bonds and nonpolar interactions with the Gly863, Ala898, Ser904, and Tyr907 residues.

Conserved residues such as His862, Tyr907, Tyr896, Ala868, Arg878, Asp766, and Gly863 play essential roles in the interaction between the inhibitors and PARP-1. Notably, Ferraris et al.^34^ have identified Gly863 and Tyr907 among these amino acids as crucial residues in inhibitors–PARP-1 interactions. Gly863 contributes to a hydrogen-bonding network, while Tyr907 engages in pi–pi stacking interactions. Notably, Tyr907, although not involved in the olaparib–PARP-1 interaction, is significant in the interaction between ZINC67913374 and PARP-1. Additionally, residues such as Ser904, Phe897, Ala898, Glu763, Leu877, Ile872, Met890, and Gly888 interact with PARP-1 through hydrophobic contacts. The critical residues Glu763, Asp766, Tyr896, Ser904, and Tyr907 are essential for the binding interaction in both PARP-1–inhibitor complexes, indicating similarities between ZINC67913374 and olaparib in their binding profiles with PARP-1.^35^

### 3.3 Cytotoxicity

Our study revealed that the molecules we have identified as potential PARP-1 inhibitor candidates effectively interact with the PARP-1 target by leveraging the specific amino acids documented in the literature. These discoveries not only validate established scientific insights but also highlight the promising prospects of our molecules, compounds **5**, **9**, and **13**, as PARP-1 inhibitors. Therefore, based on their observed interactions and potential therapeutic efficacy as potent PARP-1 inhibitors, we identified compounds **5**, **9**, and **13** as promising candidates for anticancer studies. As a result of viability experiments, compound **3** showed high cytotoxicity to all cell lines, except for MDA-MB-231 cells. Compound **3** (0.08 μg/mL) and compound **5** (1.72 μg/mL) exhibited high cytotoxicity on SH-SY5Y cells and compound **11** (2.72 μg/mL) on MCF7 cells (Table 3).

**Table 3:** IC_50_ Values of the Newly Synthesized Compounds in Different Cell Lines

	IC_50_^b^					
cell line	3	5	7	9	11	13
HaCaT	0.11 ± 0.01^a^	1.96 ± 0.25	0.15 ± 0.02	1.29 ± 0.06	3.29 ± 1.35	2.03 ± 0.08
HepG2	0.22 ± 0.04	3.43 ± 0.49	0.48 ± 0.04	5.65 ± 1.15	4.85 ± 1.31	6.53 ± 0.97
SH-SY5Y	0.08 ± 0.02	1.72 ± 0.27	0.59 ± 0.02	8.23 ± 0.35	8.23 ± 1.13	10.27 ± 0.63
A549	0.45 ± 0.08	9.31 ± 0.98	>2.5 ± 0.07	12.30 ± 0.06	9.22 ± 2.33	13.96 ± 2.39
MCF7	0.38 ± 0.07	5.64 ± 1.47	0.38 ± 0.02	1.71 ± 0.25	2.72 ± 0.27	3.40 ± 0.85
MDA-MB-231	0.46 ± 0.05	3.43 ± 0.49	0.36 ± 0.03	7.01 ± 1.47	10.30 ± 2.58	9.45 ± 0.43

a Data are represented as mean ± standard deviation.

b IC_50_ values expressed as μg/mL.

Selectivity index (SI), showing the selectivity of cytotoxicity against cancerous cell line, was calculated for the chemicals. SI should be at least more than 1, which means that cytotoxicity to cancer cells is greater than cytotoxicity to normal cells. SI was found to be “>1” for compound **3** (SI = 1.33) and compound **5** (SI = 1.14) on the SH-SY5Y cell line, and compound **11** (SI = 1.21) on the MCF7 cell line.

The results from the study, therefore, further legitimize the predictive nature of the *in silico* approach in a biological context and, at the same time, vouch for a multiomics integrated approach at the preclinical stage in drug development. This will, therefore, be an approach that is much more effective in the screening and selection of the most promising therapeutic agents—a development that would be a major advance for targeted cancer therapy.^36,37^ Further studies have also cited other predictive successes that report the highlighted potential of such in silico models toward an enhanced drug development therapeutic targeting.^38,39^

In comparison, reports in the literature for such similar naphthoquinone derivatives have not shown this clear selectivity profile^40^ but rather mentioned the piperazine substitution as the point of novelty with respect to the enhancement of specificity toward PARP-1.^41^ Taken together, these results suggest that the structural changes brought about by us in our compounds may be crucial for defining the interaction dynamics and binding efficiencies of the ligands. These attributes are eminent, ensuring selective action of this kind of compound, similar to the findings in other studies on various derivatives.^42,43^ The derivatives designed in this study for substitution by piperazine taken together in the work present encouraging inhibitory activity toward PARP-1 with considerable selectivity and potency. This further supports promising potential for the class of compounds as targeted cancer therapeutics and sets the stage for further optimization and development toward PARP-1-specific inhibitors.^44^ This further supports their promising potential as targeted cancer therapeutics and sets the stage for further optimization and development of PARP-1-specific inhibitors. This is further supported by the recent successes in targeting PARP-1 with novel inhibitors.^45^

## 4 Conclusions

The current study deals with the design and synthesis of piperazine-substituted 2,3-dichloro-5,8-dihydroxy-1,4-naphthoquinone derivatives and evaluation of their PARP-1 inhibitory activity via silico molecular docking and molecular dynamics studies. Remarkably, the newly synthesized compounds labeled as compounds **5**, **9**, and **13** have demonstrated significant in silico results regarding target specificity and binding affinity with PARP-1 docking scores of −7.17, −7.41, and −7.37 kcal/mol, respectively, and MM/GBSA scores of −52.51, −43.77, and −62.87 kcal/mol, respectively, while interacting with amino acids known to be crucial for PARP-1 inhibition. These findings underscore the importance of these new compounds as potential PARP-1 inhibitors for targeted cancer therapies. The viability data suggest that compound **5** for the SH-SY5Y cell line and compounds **9** and **13** for the MCF7 cell line exhibit higher potential utility. These novel compounds hold promise as potential PARP-1 inhibitors for the development of targeted therapeutics against cancer.
